# Comprehensive analysis of endoplasmic reticulum-related and secretome gene expression profiles in the progression of non-alcoholic fatty liver disease

**DOI:** 10.3389/fendo.2022.967016

**Published:** 2022-08-12

**Authors:** Rong Gao, Jin Wang, Xuemin He, Tongtong Wang, Li Zhou, Zhitao Ren, Jifeng Yang, Xiaoxin Xiang, Shiyi Wen, Zhuojun Yu, Heying Ai, Yuchan Wang, Hua Liang, Shasha Li, Yan Lu, Yanhua Zhu, Guojun Shi, Yanming Chen

**Affiliations:** ^1^ Department of Endocrinology and Metabolism, Guangdong Provincial Key Laboratory of Diabetology, The Third Affiliated Hospital of Sun Yat-sen University, Guangzhou, China; ^2^ Department of Clinical Immunology, The Third Affiliated Hospital of Sun Yat-sen University, Guangzhou, China

**Keywords:** weighted gene co-expression network analysis, non-alcoholic fatty liver disease, endoplasmic reticulum, progression, biomarker, secretome

## Abstract

Endoplasmic reticulum (ER) is the principal organelle for protein synthesis, such as hepatokines and transmembrane proteins, and is critical for maintaining physiological function. Dysfunction of ER is associated with metabolic disorders. However, the role of ER homeostasis as well as hepatokines in the progression of non-alcoholic fatty liver disease (NAFLD) remains to be elucidated. Here we comprehensively analyzed the RNA-seq profiles of liver biopsies from 206 NAFLD patients and 10 controls from dataset GSE135251. The co-expression modules were constructed based on weighted gene co-expression network analysis and six co-expression modules were identified, of which brown module stood out to be significantly associated with fibrosis stage and NAFLD activity score (NAS). Subsequently, cytoscape with cytoHubba plugin was applied to identify hub genes in the brown module. GO and KEGG enrichment analysis of the top 20 hub genes were performed and showed the involvement of extracellular matrix formation, collagen synthesis and decomposition, etc. Further, the expression of the top 20 hub genes were found to be a consistent increasing trend as the fibrosis stages and NAS increased, which have been validated both in HFD fed and HFHC fed mice. Among these genes, *THY1*, *PTGDS*, *TMPRSS3*, *SPON1*, *COL1A2*, *RHBDF1*, *COL3A1*, *COL5A1*, *COL1A1* and *IGFBP7* performed well in distinguishing fibrosis stage, while *COL1A2*, *COL3A1*, *THY1*, *RHBDF1* and *COL1A2* exhibited good capacity to discriminate NAS. Besides, *RHBDF1*, *COL3A1*, *QSOX1*, *STING1*, *COL5A1*, *IGFBP7*, *COL4A2*, *COL1A1*, *FKBP10* and *COL1A2* also showed a strong power in the diagnosis of NAFLD. In addition, *COL1A1*, *COL1A2*, *COL3A1*, *COL8A2*, *IGFBP7*, *PGF*, *PTGDS*, *SPON1*, *THY1* and *TIMP1* were identified as secretome genes from the top 20 hub genes. Of them, circulated *THY1* and collagen III level were validated to be significantly elevated in the MCD diet-induced mice. Thus, we provided a systemic view on understanding the pathological roles and mechanisms of ER as well as secretome in NAFLD progression. *THY1*, *COL1A1*, *COL1A2*, *COL3A1* and *RHBDF1* could be served as candidate biomarkers to evaluate the progression of NAFLD.

## Introduction

In tandem with the global obesity epidemic, the incidence of non-alcoholic fatty liver diseases (NAFLD) is undergoing a steep increment and becoming one of the most common chronic liver diseases, with an estimated prevalence of 25.24% in adults worldwide (1). Patients with NAFLD are associated with an increasing risk for obesity, diabetes, insulin resistance and adverse cardiovascular outcomes, imposing a heavy economic burden on individuals and society (2).

NAFLD is generally considered to be a hepatic manifestation of the metabolic syndrome with highly heterogeneous clinical features, which encompasses a spectrum of conditions, ranging from simple hepatic steatosis (NAFL) to nonalcoholic steatohepatitis (NASH); some progress to cirrhosis and ultimately hepatocellular carcinoma (HCC) (3). Hence, NAFLD is thought to be a progressive disease. The majority of patients exhibit a stable stage of simple hepatic steatosis without evidence of hepatocellular injury, while about 20% patients progress to NASH as featured by inflammation, hepatocyte ballooning and fibrosis on the basis of fat accumulation. The speed of NAFLD progression varies among individuals. Nevertheless, the detailed underlying pathogenic mechanisms of transversion from NAFL to NASH remains ambiguous. Currently, it is thought to be the interplay between various factors such as environment, microbiome, epigenetics, susceptibility gene polymorphism and metabolism in the progression of NAFLD (4, 5). The “two-hit” hypothesis is a widely accepted hypothesis for the pathogenesis of NAFLD. The “first hit” is considered as the accumulation of hepatic triglycerides, generally secondary to a high-fat diet, obesity and insulin resistance, which contributes to the initiation of NAFLD. The “first hit” makes it more susceptible to further insults by stress, which are recognized as the “second hit”, such as endoplasmic reticulum (ER) stress, oxidative stress, accumulation of mitochondrial dysfunction, accumulation of inflammatory cytokines and apoptosis. The second hits would promote hepatic inflammation, necrosis and fibrosis, contributing to the progression from NAFL to NASH (6).

ER is the principal organelle responsible for a variety of cellular processes, including protein synthesis, folding and secretion, lipid and glucose metabolism, and intracellular calcium storage (7). Liver acts as an essential secretory organ with abundant endoplasmic reticulum to orchestrate protein homeostasis and the systematic metabolic homeostasis (8, 9). Under chronic metabolic stress hepatocytic ER serves as a double-edged sword to handle the disturbance of ER environment (10). On the one hand, the ER in hepatocytes possess powerful capacity to restore ER homeostasis and maintain the critical metabolic functions of liver by attenuating protein translation, increasing chaperones and alleviating the burden of misfolded/unfolded proteins. On the other hand, the low-grade but persistent ER stress will cause ER dysfunction and inappropriately regulate expression of an array of molecules, consequently leading to inflammatory activation, insulin resistance, hepatocyte apoptosis, and ultimately derangement the architecture and function of liver (11–13). Liver acts as an endocrine organ to communicate with other metabolic tissues *via* hepatokines. The secretion pattern of hepatokines might be perturbed in response to metabolic stress, resulting in the aggravation of NAFLD. The current research on hepatic ER stress and NAFLD are mostly focus on specific molecular mechanisms, while the underlying mechanism from a whole-transcriptome perspective into understanding the role of ER in NAFLD progression remains to be explored.

In this study, we constructed the co-expression network in ER related gene sets based on weighted gene co-expression network analysis (WGCNA) and explored the association between interested modules and clinical traits. We identified hub genes as well as secretome genes as candidate biomarkers to evaluate the severity of NAFLD, providing a comprehensive view on the essential role of ER during the progression of NAFLD.

## Methods

### Animals studies

16-week-old ob/m and ob/ob mice were acquired from Jackson Laboratories and livers were harvested under fasted state for RNA sequencing. The 8-week-old C57BL/6 male mice were fed with a normal chew diet or HFHC diet (containing 40% fat, 22% fructose, and 2% cholesterol) for 28 weeks as described previously (14). Livers were harvested for quantitative real-time PCR (qRT-PCR) and western blot. The 8-week-old C57BL/6 male mice were fed with a normal chew diet or MCD diet (containing 40% sucrose and 10% fat, lacking methionine and choline) for 4 weeks as described previously (15), serum was collected for western blot to determine the level of hub genes encoded secreted proteins. All animal experiments in this study were approved by the Ethics Committee of Sun Yat-sen University.

### Data collection, preprocessing and selection of gene sets

The expression profiling of high throughput RNA sequencing dataset GSE135251 with clinical traits were downloaded from GEO database in NCBI. In this dataset, a total of 216 samples obtained from snap frozen liver biopsies, including 206 NAFLD participants with different fibrosis stages and NAS, as well as 10 control cases were studied (16). We converted raw counts to FPKM with GenomicFeatures R package.

To obtain the ER related gene sets, hands-on search was conducted based on keywords “endoplasmic reticulum”, “ER” or “protein quality control” and 22 related gene sets were acquired from Molecular Signature Database v7.4 (MSigDB) (17) and Kyoto Encyclopedia of Genes and Genomes (KEGG) pathway database. A total of 2397 genes were obtained after removing the overlap genes (**Figure S1A**). After filtering low abundance expression genes (more than half the samples in one group with FPKM less than 0.5), we acquired 1231 genes for further analysis (**Supplementary Table 1**). Meanwhile, secretome database containing 1,378 genes were acquired from PUBMED (18).

To explore the consistency among samples in ER related genes, we performed sample hierarchical clustering using the R “hclust” function and found one obvious outlier (GSM3998294), which was excluded from the subsequent analysis. In the end, a total of 215 participants with clinical data were included in the study (**Figure S1B**).

The microarray dataset GSE28619 including 15 liver samples with alcoholic hepatitis and 7 normal liver samples from human were obtained from GEO database (19). Chip probeset annotations and differentially expression analysis were conducted by GEO2R.

### Co-expression network construction and module identification

R package “WGCNA” (20) was used with ER related genes for network construction. The “pickSoftThreshold” function in WGCNA was used to calculate the soft threshold. Six modules were successfully constructed by dynamic tree cutting using the “blockwiseModules function” in WGCNA with a merging threshold of 0.25, a minimum module size of 30 and deepSplit of 3. The adjacency matrix was transformed into a topology overlay matrix (TOM) and the correlation between each module was detected by cluster analysis. Subsequently, the Pearson correlation coefficient between module eigengenes and clinical traits were calculated and visualized by heatmap. Volcano plot and principal component analysis (PCA) plot were performed to visualize the relationship between the interested module and phenotype using “GraphPad Prism 8.0” and “fviz_pca_ind” function of factoextra package respectively.

### The protein-protein interaction (PPI) network construction and hub genes screening

Edge and node information of the interested module were exported using “exportNetworkToCytoscape” function in WGCNA (threshold ≥ 0.5) to construct PPI network and visualized with Cytoscape software. The hub genes of PPI network were analyzed using cytohubba (21) plugin with 11 algorithms (MCC, DMNC, MNC, Degree, EPC, EcCentricity, Closeness, BottleNeck, Radialit, Betweenness, Stress and ClusteringCoefficient) and visualized in the PPI network calculated by MCC with Cytoscape.

### Gene ontology (GO) and kyoto encyclopedia of genes and genomes (KEGG) enrichment analysis

GO and KEGG enrichment analysis with the top 20 hub genes were performed using WebGestalt (http://www.webgestalt.org/option.php) online and visualized with Sangerbox tools (http://www.sangerbox.com/tool).

### Literature search of hub genes

Literature mining was conducted by database searching using GenCLiP 3.0 (22) (http://cismu.net/genclip3/analysis.php) and hands-on searching with keywords, citation searching and reference list checking.

### RNA isolation and quantitative real-time PCR

RNA isolation and qRT-PCR were performed according to the manufacturer’s instructions. Briefly, total RNA of livers from normal and HFHC diet mice were isolated using TRIZOL (Invitrogen, CA) method and cDNA was synthesized using Evo M-MLV Mix Kit with gDNA Clean (Accurate biology, China). Subsequently, qRT-PCR with cDNA was conducted using an SYBR Green reagent (Accurate biology, China) on a QuantStudio5 Real-Time PCR System (Applied biosystems, Singapore). Quantitative PCR data was analyzed according to the 2^-△△^Ct method. The primer sequences were available in **Supplementary Table 2**.

### RNA sequencing

High throughput RNA sequencing of livers from *ob/m* and *ob/ob* mice was performed on Illumina HiSeq 4000 by Shanghai Applied Protein Technology as previously described (23). Differential expression analysis was performed by DESeq2 and the criterion for differentially expressed genes was *P* value < 0.05.

### Western blots and antibodies

Livers were harvested and lysed in lysis buffer (containing 50mM Tris-HCl pH 8.0, 1mM EDTA, 1% TritonX-100,150 mM NaCl [pH 7.5], supplied with 1 mM DTT and protease inhibitor before use) and placed on ice for 20 min. After centrifuging at 12,000g for 10min, supernatants were mixed with sample buffer (containing 250 mM Tris-HCl [pH 6.8], 0.1% SDS, 1.44M β-mercaptoethanol, 50% glycerol and 0.05% bromophenyl blue). Lysates were boiled at 95° for 5-10 min and mixed fully prior to be equally subjected to SDS-PAGE gel.

Antibody used in the study were: *THY1*(Proteintech, 1:1000),collagen I (Servicebio, 1:1000), collagen III (Novus, 1:1000), STING (Cell Signaling Technology, 1:1000), ATF4 (Cell Signaling Technology, 1:2,000), BIP (Abcam, 1:5,000), HSP90 (Cell Signaling Technology, 1:2,000), HRP conjugated goat anti-mouse secondary antibody (Biorad, 1706516, 1:5,000), HRP conjugated goat anti-rabbit secondary antibody(Biorad, 1706515, 1:5,000). Band density was photographed and quantified using the Image Lab software on the ChemiDOC XRS+ system (Bio-Rad).

### Statistical analysis

Statistical significance was tested by Two-tailed Student’s t test for two group comparisons. One-way ANOVA was used for multiple comparisons with Bonferroni correction. Receiver operating characteristic (ROC) curves were obtained using the R package pROC (version 1.17.0.1) with the top 20 hub genes respectively. Data were expressed as means ± standard error of the mean (SEM). *P* value < 0.05 was considered to be statistically significant.

## Results

### Identification of key modules with ER related genes based on WGCNA

**Figure 1:**
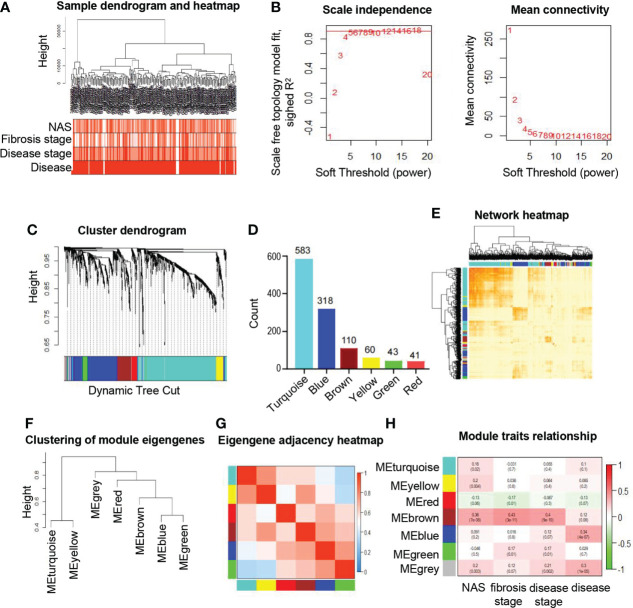
Identification and characteristic of key modules in ER related genes based on WGCNA. **(A)** Hierarchical clustering dendrogram of the samples and clinical traits. The color intensity is proportional to the properties of clinical traits including NAS (0-8), fibrosis stages (F0-F4), disease stages (control, early and moderate) and disease (NAFLD or not). **(B)** Soft threshold selection process to obtain the scale-free fit index of network topology. **(C)** Hierarchical cluster tree shows six key modules based on WGCNA. The leaves in the tree represent genes and the colors underneath the dendrogram represent co-expression module determined by the dynamic tree cut. **(D)** Number of genes in each module. **(E)** Interaction of co-expression of genes based on TOM in the analysis. The progressively darker color in the heatmap represents higher topological overlap. Hierarchical clustering dendrogram **(F)** and heatmap **(G)** show the relationship among the six module eigengenes. Close branches of the dendrogram are highly correlated. In the heatmap, each row and column correspond to one module eigengene. Blue color represents low adjacency, while red represents high adjacency. **(H)** Correlation of module eigengenes and clinical traits. Each row represents module and each column represents corresponding clinical traits. Pearson correlation coefficients and *P* values are displayed and the boxes are colored according to the Pearson correlation coefficients. WGCNA, weighted gene co-expression network analysis; NAFLD, non-alcoholic fatty liver disease; TOM, topological overlap matrix.

To obtain the ER related gene sets, we downloaded 22 related gene sets from MsigDB and KEGG pathway database, and 1231 genes were selected for further analysis (**Figure S1A**) as described in the methods. To explore the consistency among samples in ER related genes, a total of 215 participants with clinical data were included in the study from public database without sequencing data GSM3998294 (**Figure S1B**) as described in the methods. The sample dendrogram and heatmap of clinical characteristics were combined to show an obvious association of clinical traits and clustered samples (**Figure 1A**). To construct co-expression network with ER related genes based on WGCNA, we first calculated the soft threshold to determine the candidate power with scale free topology model fit (R^2^) close to 0.9 with a relatively high mean connectivity. We selected soft threshold power as 5 (R^2^ = 0.89) to construct an approximate scale-free network (**Figure 1B**). As shown in **Figure 1C**, a total of six co-expression modules with similar expression patterns were identified based on the dynamic tree-clipping algorithm. The gene counts in each module ranged from 41 (red module) to 583 (turquoise module) (**Figure 1D**). In addition, a gene set that could not be clustered into any of the modules were labeled as gray, which would be excluded in the following analysis. The interactions of the identified modules were visualized by the heatmap of topological overlap matrix (TOM) in the gene network and we found genes from the same modules were highly relevant, while a relatively independent gene expression among modules (**Figure 1E**). Furthermore, module eigengenes of each module were calculated to elucidate the relationships among modules. As shown in the eigengene adjacency dendrogram and heatmap (**Figures 1F, G**), different degrees of interactive connectivity indicated different characteristics and functions among modules. Taking together, these data demonstrated that a co-expression network of six modules with ER related genes was successfully constructed using WGCNA.

### Association of the selected module with the progression of NAFLD

**Figure 2:**
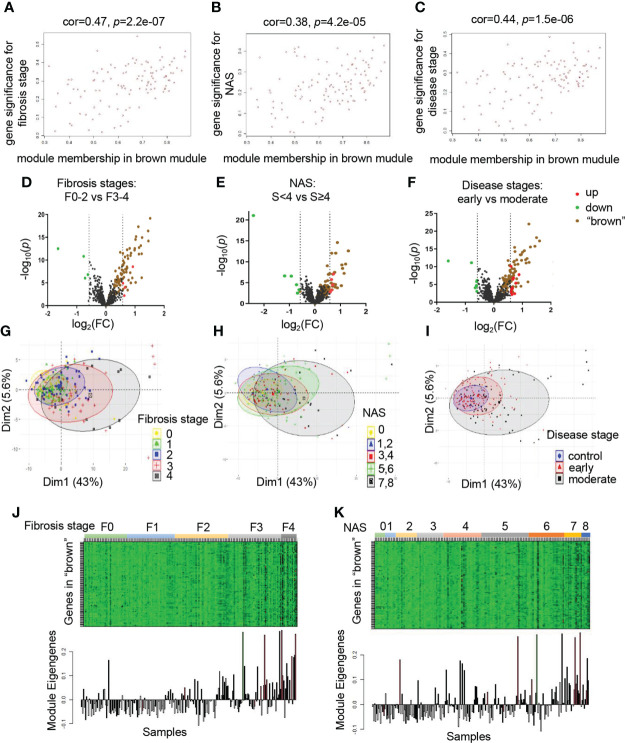
The brown module is significantly correlated with clinical traits. **(A–C)** Correlation between MM in brown module and GS for fibrosis stages (F0-F4) **(A)**, NAS (0-8) **(B)**, disease stages (control, early and moderate) **(C)** are calculated. Corresponding Pearson correlation coefficients and *P* values are displayed. **(D–F)** Volcano plots to visualize differential expression patterns between groups based on fibrosis stages, NAS and disease stages, respectively. Genes up-regulated (FC>1.5, *p* value <0.05) are depicted in red dots and those down regulated (FC<0.66, *p* value <0.05) are depicted in green dots. All the genes from brown module are shown in brown dots. The rest of genes without significant difference are shown in black dots. **(G–I)** PCA analysis of the genes from brown module. The points represent samples based on the top two principal components of gene expression profiles. The colors represent groups of samples based on fibrosis stages, NAS and disease stages, respectively. **(J, K)** The relationship of gene expression feature of brown module and fibrosis stages, NAS, respectively. The heatmaps represent gene expression of brown module in each sample. The bar graphs represent the module eigengenes of brown module in the corresponding samples. GS, gene significance; MM, module membership; PCA, principal component analysis.

To evaluate the relationship between modules and clinical traits, the Pearson’s correlation coefficients linking module eigenvalues and clinical features were calculated. As shown in **Figure 1H**, the brown module exhibited significant positive correlations to several clinical traits, including fibrosis stages (r=0.43, *p*=3e-11), NAS (r=0.36, *p*=7e-8) and disease stages (r=0.4, *p*=9e-10). These features are important standards to assess the severity of inflammation and steatosis in NAFLD, which represent the progression of NAFLD (24). In addition, the blue module was significantly associated with respective types of diseases (r=0.34, *p*=4e-7). Subsequently, the significant correlations between the module eigenvalues and traits were further conformed by intramodular analysis. The scatter plots revealed a significant positive connection between module membership (MM) of genes in brown and gene significance (GS) for fibrosis stages (r=0.47, *p*=2.2e-07) (**Figure 2A**), NAS (r=0.38, *p*=4.2e-05) (**Figure 2B**) and disease stages (r=0.44, *p*=1.5e-06) (**Figure 2C**), suggesting that the genes in brown module, especially those with both high MM and GS, might be of great importance on the progression of NAFLD (**Figures 2A–C**).

To provide further evidence for the relationship of brown module and clinical stages in detail, we divided patient groups into dichotomies based on clinical traits, including fibrosis stages (F0-F2 vs F3-F4), NAS (<4 vs ≥4), disease stages (early vs moderate), respectively. We investigated whether the genes in brown module existed prominent difference between clinical subgroups. As depicted in the volcano plots, a large number of ER related genes altered significantly between groups, regardless of divided by fibrosis stages, NAS, or disease stages, suggesting the involvement of ER in the progression of NAFLD. Thus, we explored the expression pattern of brown module by highlighting genes in the brown plots. We found that majority of these genes exhibited varying degree of up-regulation. Of note, these genes also accounted for most of the significant upregulated genes in different disease groups, indicating that the brown module might be the most critical component to make a difference (**Figures 2D–F**). Furthermore, PCA analysis was performed to reveal that the global genetic characteristics of brown module gradually vary with the increasement in the clinical rating (**Figures 2G–I**). In the heatmap, the gene expression levels showed an increased tendency in samples with the progression of NAFLD (**Figures 2J, K**). Taken together, these data demonstrated that the brown module was identified as the key module, indicating that those genes probably played essential roles in the progression of NAFLD. Therefore, we chose the brown module for the further analysis.

### Identification of hub genes in the brown module

**Figure 3:**
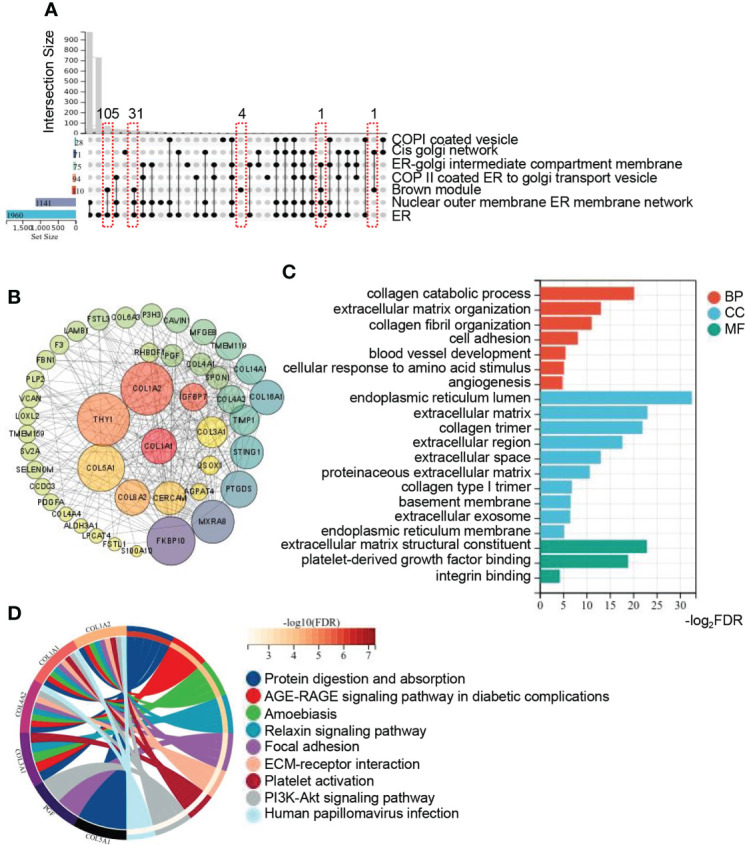
Identification of hub genes in brown module and function annotation. **(A)** Upset plot summarizes the intersection among ER related gene sets from GO_CC and genes from brown module. The bar plot in the left represents the gene number of each set. The lines in red frames represent the number of intersection genes of the brown module and other gene sets. **(B)** Network of genes with the weight (TOM) more than 1.0 in the brown module. Genes in the central highlighted red to green are the top 20 hub genes calculated by MCC. Both the color and size of the circle are proportional to the intramodular connectivity. **(C, D)** GO and KEGG pathway enrichment analysis of the top 20 hub genes from brown module. Circos plot is to indicate the relationship between genes and KEGG terms. ER, endoplasmic reticulum; GO, gene ontology; BP, biological process; CC, cellular component; MF, molecular function; KEGG, kyoto encyclopedia of genes and genomes.

To analyze the intracellular localization of the brown module genes and their possible roles involving in the progression of NAFLD, 6 ER related gene sets acquired from GO “cellular component”, including ER and ER associated crosstalk with other organelles (Golgi, nuclear, membrane, etc), were intersected with genes of the brown module. The upset plot revealed that majority of genes were located in the ER (**Figure 3A**). To explore the hub genes in the brown module, Cytoscape was utilized to analyze the core subnetworks of brown module and finally identified key driver genes *via* the degree method with cytoHubba plugin. A PPI network was established and colored by degree to visualize the interactions, in which the top 20 hub genes were shown at the center of the network, including type I collagen alpha 1 (*COL1A1*), insulin-like growth factor-binding protein 7 (*IGFBP7*), type I collagen alpha 2 (*COL1A2*), thymocyte differentiation antigen 1 (*THY1*), Type VIII collagen alpha 2 (COL8A2), cerebral endothelial cell adhesion molecule (*CERCAM*), type V collagen alpha 1 (*COL5A1*),acylglycerophosphate acyltransferase 4(*AGPAT4*), quiescin sulfhydryl oxidase homolog (*QSOX1*), stimulator of interferon genes 1(*STING1*), Rhomboid family-1 gene (*RHBDF1*), transmembrane protease serine 3(*TMPRSS3*), type IV collagen alpha 2 (*COL4A2*), Spondin 1(*SPON1*), placental growth factor (*PGF*), type III collagen alpha 1 (*COL3A1*), FK506 binding protein 10( *FKBP10* ), prostaglandin D2 synthase (*PTGDS*), matrix remodeling associated 8(MXRA8) and metallopeptidase inhibitor 1(*TIMP1*)(**Figure 3B**). To investigate the functional distribution of these hub genes and pathway enrichment, GO and KEGG pathway analysis was performed with the top 20 hub genes. As summarized in **Figure 3C**, biological processes of GO were mainly involved in collagen and fiber associated processes, including collagen catabolic process, extracellular matrix organization, collagen fibril organization, cell adhesion, etc. Cellular components of GO major demonstrated obvious enrichment of endoplasmic reticulum lumen, extracellular matrix, collagen trimer, etc. Molecular functions of GO were prominent involved in extracellular matrix structural constituent, platelet-derived growth factor binding and so on. Meanwhile, KEGG signaling pathway were significantly enriched in protein digestion and absorption, AGE-RAGE signaling pathway in diabetic complications, focal adhesion, ECM-receptor interaction, PI3K-Akt signaling pathway, etc. (**Figure 3D**). These pathways were apparently involved in the process of fibrosis, inflammation and steatosis, providing further evidence for ER to be vital in participating the progression of NAFLD.

### The expression pattern of hub genes and their diagnostic value in the NAFLD progression

**Figure 4:**
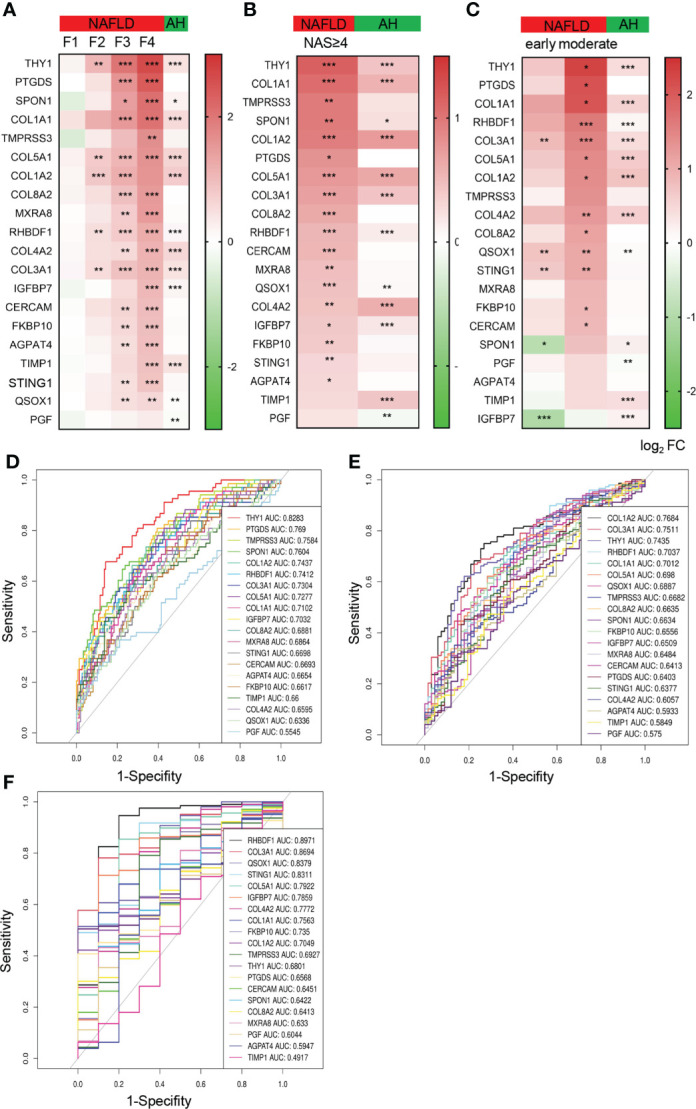
The expression pattern of the top 20 hub genes and their diagnostic value in the NAFLD progression. **(A–C)** Heatmap of the relative expression of top 20 hub genes in NAFLD and AH database, grouped by fibrosis stages (F0-F4) **(A)**, NAS (NAS<4 and NAS≥4) **(B)**, disease stages (control, early and moderate) **(C)**. The colors represent log_2_fold change determined by RNA sequencing (NAFLD database) or chip array (AH database). Data are represented as means ± SEM. *P* value was adjusted in **(A)**. *P < 0.05, **P< 0.01 and ***P< 0.001. **(D–F)** ROC curves show the specificity and sensitivity of the 20 hub genes as biomarkers to recognize fibrosis stage of NAFLD (F<2 or ≥2) **(D)**, NASH (NAS <4 or ≥4) **(E)** and NAFLD **(F)**. ROC, receiver operating characteristic; AUC, area under the curve, AH, alcoholic hepatitis.

To further illustrate the expression pattern of the hub genes and tap into their potential application in the progression of NAFLD, we extracted the top 20 hub genes ranked by MCC from the gene expression list to analyze their relative levels in groups with varying extents of NAFLD. Of note, the expression of these identified hub genes showed an increasing tendency as the fibrosis degree increases (**Figure 4A**). Compared with those of control, most the expression changes were found to be significant on the stage of F3 and F4, indicating the possibility to use F3 as a cut-off to evaluate the severity of fibrosis in NAFLD. In addition, we also measured the expression pattern of hub genes according to NAS and disease stages respectively, similar increasing trends were exhibited as shown in **Figures 4B, C**. Considering the similarities in pathology between alcoholic fatty liver and non-alcoholic fatty liver, a dataset of liver samples from normal and alcoholic hepatitis patient (AH) were used as another control group. A significant elevation of nearly half of the hub genes (including *THY1*, *SPON1*, *COL1A1*, *COL5A1*, *COL1A2*, *RHBDF1*, *COL4A2*, *COL3A1*, *IGFBP7*, *TIMP1*, *QSOX1* and *PGF*) could be observed both in AH and NAFLD in comparison with the normal control, while *PTGDS*, *TMPRSS3*, *COL8A2*, *MXRA8*, *FKBP10* , *CERCAM*, *AGPAT4* and *STING1* did not altered significantly in AH but increased prominently in the NAFLD progression (**Figures 4A–C**), indicating the critical role of the identified hub genes in both NAFLD and AH. The data also gave us clues to explore the potential biomarkers to distinguish NAFLD and AH.

Among the hub genes, we found that *COL1A1*, *COL1A2*, *COL3A1*, *COL4A2*, *COL5A1*, *COL8A2* are members of fibrillar forming collagen in response to extracellular matrix formation. *IGFBP7*, *THY1*, *SPON1* and *CERCAM* are generally involved in cell adhesion and signaling. *RHBDF1* and *TIMP1* are of importance for cell migration, proliferation and inflammation. These biological processes are believed to play critical roles in hepatic fibrogenesis and steatosis. Hence, we speculated that these hub genes might be of value in the diagnosis of NAFLD severity. Thus, the ROC analysis was conducted to evaluate the specificity and sensitivity of the top 20 hub genes for recognition of NAFLD severity. Meanwhile, the area under the curves (AUC) were calculated to assess the performance of the ROC curves. As shown in **Figure 4D**, *THY1* performed best among the genes to distinguish fibrosis F0-F2 to F3-F4 with AUC more than 0.8, while *PTGDS*, *TMPRSS3*, *SPON1*, *COL1A2*, *RHBDF1*, *COL3A1*, *COL5A1*, *COL1A1* and *IGFBP7* also performed well. Similarly, *COL1A2*, *COL3A1*, *THY1*, *RHBDF1* and *COL1A2* exhibited good value for discriminating groups between NAS<4 and NAS≥4 (**Figure 4E**). In addition, the value of these genes in diagnosing NAFLD were also speculated and we found that *RHBDF1*, *COL3A1*, *QSOX1* and *STING1* showed a significant diagnosis power with AUC more than 0.8, while *COL5A1*, *IGFBP7*, *COL4A2*, *COL1A1*, *FKBP10* and *COL1A2* also came out well with AUC more than 0.7 (**Figure 4F**). These data demonstrated that the hub genes identified from ER related gene sets were expected to become the candidate biomarkers for the indication of NAFLD progression.

### Validation of hub genes from brown module on NAFLD mice models

**Figure 5:**
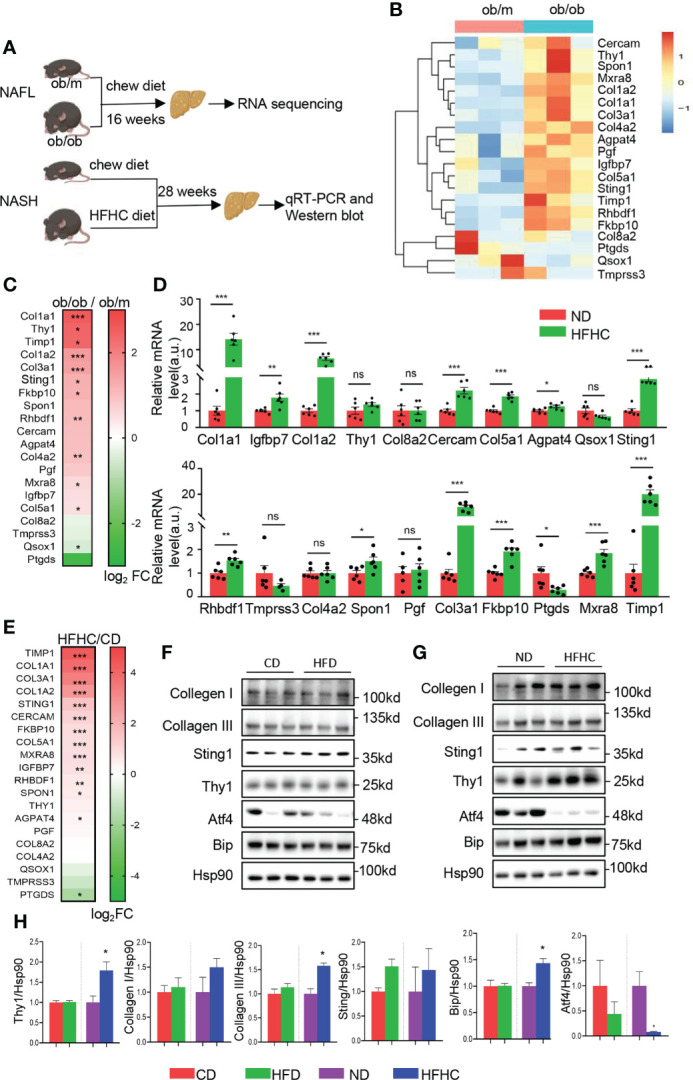
Validation of the top 20 hub genes from brown module on NAFLD mice models. **(A)**Schematic diagram of animal experiments. *Ob/m* and *ob/ob* mice were fed with chew diet for 16 weeks to establish NAFL mice model and livers were harvested for RNA sequencing. C57BL/6 mice fed with HFHC diet or normal chew diet for 28 weeks to establish NASH mice model and livers were harvested for qRT-PCR and Western blot. **(B)** The heatmap represents relative expression of the top 20 hub genes in livers of *ob/m* and *ob/ob* mice determined by RNA sequencing. **(C)** Heatmap of the top 20 hub genes of *ob/m* and *ob/ob* mice, colored by log_2_fold change determined by RNA sequencing. **(D)** mRNA level of the top 20 hub genes in livers were determined by qRT-PCR with quantification (n=6). Each experiment had 2 technical replicates. **(E)** Heatmap of the top 20 hub genes of HFHC-fed and control mice, colored by log_2_fold change determined by qRT-PCR. **(F–H)**. The protein level of top ranked hub genes identified by ROC analysis were determined in NAFL, NASH and corresponding control mice by Western blots **(F, G)** with quantification (n=3) **(H)**. Data are represented as means ± SEM. *P < 0.05, **P< 0.01 and ***P< 0.001. ns, not significant; ROC, receiver operating characteristic; HFD, high fat diet mice; CD, the control for HFD mice; HFHC, high-fat and high-cholesterol diet mice; ND, the control for HFHC mice. ns, not significant.

To validate the expression of the top 20 hub genes in the progression of NAFLD, NAFL model of leptin knockout (*ob/ob*) and control (*ob/m*) mice fed with chew diet for 16 weeks and NASH model of C57BL/6 mice fed with HFHC diet or normal chew diet for 28 weeks were established respectively (**Figure 5A**). Livers of *ob/m* and *ob/ob* mice were harvested for RNA sequencing and we found that most of the identified hub genes exhibited an elevated trend (**Figures 5B, C**). Meanwhile, we confirmed the hub genes of livers from NASH and corresponding control mice by qRT-PCR and found that **COL1A1*, *IGFBP7*, *COL1A2*, Cercam, *COL5A1*, Agpat4, *STING1*, *RHBDF1*, *SPON1*, *COL3A1*, *FKBP10* , Mxra8* and *TIMP1* were significantly increased in HFHC fed mice, while *PTGDS* was reduced in the NASH group in comparison with the control group (**Figures 5D, E**).

To further explore the level of hub genes on protein level, western blot analysis of the top ranked hub genes with high AUC in ROC analysis were performed in HFD and HFHC-fed mice, as well as the corresponding control mice. As shown in **Figures 5F, H**, the expression of **THY1*, *STING1**, *collagen I* and *III* did not altered significantly in HFD mice. The expression of *THY1* and *Collagen III* exhibited significant elevation in HFHC-fed mice, while the level of *STING1* and *collagen I* did not altered significantly in comparison with corresponding control (**Figures 5G, H**). Meanwhile, the prominent alteration of several ER stress markers (Atf4 and Bip) in HFD and HFHC-fed mice (**Figures 5F–H**) indicated the disturbance of ER function in NAFLD, which gave further evidence to the critical role of ER function in the NAFLD progression. Overall, these data validate the expression pattern of the identified hub genes in mice models, providing more mechanistic insights into individual genes that involved in NAFLD disease through transgenic mouse models.

### Identification of secretome genes from brown module

**Figure 6:**
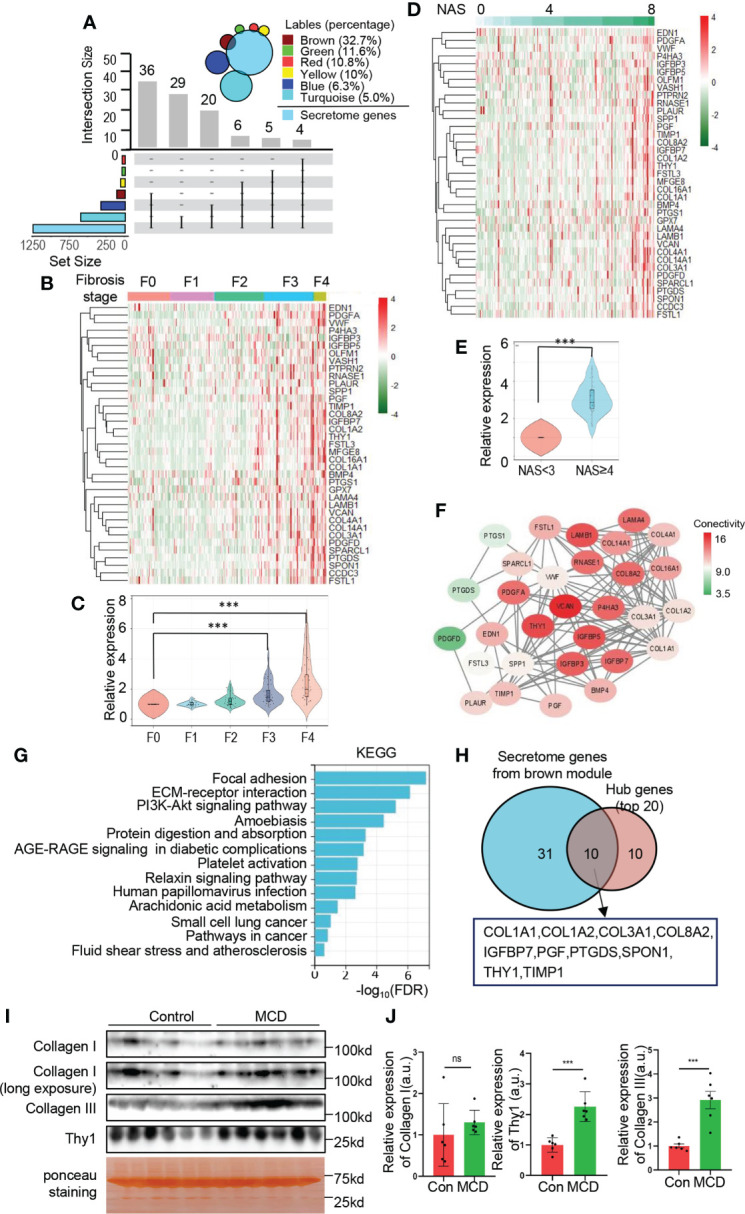
Identification of secretome genes from brown module. **(A)** Upset plot summarizes the intersection between secretome database and genes of each module. The bar plot represents the intersection gene numbers. The Venn diagram in the top right corner shows the percentage of secretome genes of each module. **(B–E)** The relative expression of secretome genes from brown module are exhibited with heatmap **(B, D)** with the corresponding quantification **(C, E)**. Heatmaps are normalized by z-score and the columns are arranged according to fibrosis stages **(B)** and NAS **(D)**, respectively. Data are represented as means ± SEM. ***adjusted *P* value < 0.001 or ***P value < 0.001 as indicated by one-way ANOVA test **(C)** or two-tailed Student’s t test **(E). (F)** Network of intersection genes between brown module and secretome database. Colors of the circle are proportional to the intramodular connectivity. **(G)** KEGG analysis with secretome genes from brown module. **(H)** Venn diagram showing intersection of the top 20 hub genes calculated by MCC and secretome genes from brown module. **(I, J)** C57BL/6 mice fed with MCD diet or normal chew diet for 4 weeks to establish NASH mice model. The protein levels of collagen I, III and Thy1 in serum were determined by Western blots **(I)** with quantification (n=6) **(J)**. Data are represented as means ± SEM. ns, not significant; ***P< 0.001.

Liver has been recognized as an endocrine organ which secretes hepatokines to play crucial roles in the regulation of systemic metabolism. We further investigated the secretome genes from brown module. As displayed in **Figure 6A**, a total of 36 genes from brown module (36/110, 32.7%) were identified as secretome genes when overlapped with the secretome database, and brown module represented the module with largest number and proportion of secretome genes. The relative expression of secretome genes from brown module showed a significantly increasing trend as the fibrosis degree and NAS increase (**Figures 6B–E**). Moreover, multiple secretome genes from brown module were predicted to interact with each other closely, as revealed by PPI network (**Figure 6F**). To further explore the potential physiological function of these secretome genes, KEGG analysis was performed and showed significant enrichment in focal adhesion, ECM-receptor interaction, PI3K-Akt signaling pathway, protein digestion and absorption, etc. (**Figure 6G**). These data suggest an important role of ER related secretome genes in orchestrating systemic metabolism. In addition, the hub genes explored in **Figure 4** were overlapped with secretome database and showed 10 out of 20 genes were secretome genes, including *COL1A1*, *COL1A2*, *COL3A1*, COL8A2, *IGFBP7*, *PGF*, *PTGDS*, *SPON1*, *TIMP1* and *THY1*(**Figure 6H**). To validate the expression of secreted proteins encoded by hub genes in circulation, serum from MCD diet induced NASH mice and the corresponding control were collected for further exploration. The expressions of *THY1* and collagen III exhibited significant elevations in the NASH mice in comparison to the control group, while collagen I did not altered significantly (**Figures 6I, J**), suggesting the possibility of these genes serving as serum biomarkers to validate the severity of NAFLD. Taken together, our data provided evidence of the important roles of ER homeostasis and secretome genes in the NAFLD progression.

### Literature search of hub genes

Finally, literature search for the relationship of the top 20 hub genes and NAFLD was conducted combining with database analysis and hands-on search. As shown in **Table 1**, the database analysis revealed that *TIMP1*, *STING1*, *COL1A1*, *IGFBP7*, *PTGDS*, *COL1A2*, *COL3A1*, *THY1* have been reported to be related to NAFLD. Among them, *TIMP1*, *STING1*, *COL1A1*, *IGFBP7*, *PTGDS*, *COL1A2* and *COL3A1* have been experimentally validated related to NAFLD on mRNA or protein level.

**Table 1:** The searching results of related literature about the top 20 hub genes and NAFLD.

Gene symbol	Hits in GenClip3	Hits in Pubmed	Hits with experimentally validated	Total
TIMP1	50	100	91	11133
STING1	11	12	9	2999
COL1A1	34	70	70	3984
IGFBP7	4	5	3	878
PTGDS	2	1	1	1206
COL1A2	8	15	11	3678
COL3A1	6	9	7	1515
THY1	1	2	0	7164

Hit, number of papers related to gene and NAFLD; Total, number of papers searched for genes.

## Discussion

In the past few decades, the mechanism underlying of NAFLD progression remains not clear, but cumulative reports have revealed that the ER and related signaling networks are emerging as an essential determinant of metabolic disorders (25). ER consists of the largest membrane system of the cytoplasm, organically connecting various organelle structures to enhance the function of protein folding and trafficking effectively. Besides, ER is the major site for the synthesis of secretory proteins (such as hepatokines) and lipids, which is vital in both physiological and pathological conditions (26). In case of NAFLD, the ER is unable to cope with the challenge of excessive caloric stress and progressively induces homeostatic systems deterioration, giving rise to an array of disease pathogenesis (27). Meanwhile, liver acts as a critical endocrine organ to secrete hepatokines and crosstalk with other metabolic organs in autocrine, endocrine, or paracrine pathways, which is closely related to normal endoplasmic reticulum function. Dysregulated endocrine function of liver plays important roles in NAFLD progression. Thus, it is appealing to investigate the role of ER and ER related hepatokines in NAFLD progression in a global perspective. Our data identified 6 clusters from ER related gene sets and corroborated in multifaceted ways that the module of interest gene set consisted of 110 genes was relevant to the clinical signatures of NAFLD progression, especially to the progression of fibrosis (**Figure 1**). Of note, the cluster of genes involved in fibrosis stages, NAS and disease stages were different from those involved in the onset of NAFLD, indicating the distinct pathogenic mechanisms during different stages of NAFLD (**Figure 2**).

In our study, we identified the hub genes and demonstrate an increasing trend in the progression of NAFLD (**Figure 4**). 8 out of top 20 hub genes have been reported to be associated to NAFLD by literature searching, including *STING1*, *COL1A1*, *IGFBP7*, *PTGDS*, *TIMP1*, *COL1A2* and *COL3A1*. Among the hub genes, *COL1A1*, *COL1A2*, *COL3A1*, *THY1*, *RHBDF1* perform particularly well in recognition of the severity of NAFLD. *COL1A1*, *COL1A2* and *COL3A1* are essential in type I and III procollagen synthesis and extracellular matrix formation in the process of fibrosis. An integral ER is required for proper folding, secretion and fibril formation of collagen (28). In the context of NAFLD, the dysfunction of ER contributes to the activation of inflammation and fatty acid oxidation, which are key drivers involved in the production of type I and III collagen (29, 30). Type I collagen is supposed to be secreted out of the cell and accumulate in the extracellular matrix, involving the process of fibrosis. Therefore, it is reasonable to suggest that *COL1A1*, *COL1A2* and *COL3A1* could link ER function and NAFLD progression. In addition, the role of type I procollagen involves in multiple organs and physiological processes, including osteoporosis, pulmonary arterial hypertension, systemic sclerosis, etc. through the intracellular or extracellular action (31). The critical function of serum type I procollagen act as cytokine to regulate systemic homeostasis remains further exploration.

Few studies of *RHBDF1*, *THY1*, MXRA8, *FKBP10* , *PGF*, *SPON1*, *COL4A2*, *TMPRSS3*, *QSOX1*, AGPAT4, *COL5A1*, CERCAM and *COL8A2* have been reported at present in the field of NAFLD. Among them, *RHBDF1* is a serine proteases localized in the ER and primarily responsible for protein quality control mechanisms by promoting ER-associated degradation (ERAD), which involving in diverse biological functions, such as growth factor signal transduction, mitochondrial morphology and inflammation (32). The abnormal expression of *RHBDF1* was reported in various diseases including cancer, systemic inflammation diseases, Alzheimer’s disease, etc (33). However, few studies were reported on metabolic deceases. Our data provided evidence for the potential role of RHBDF in NAFLD progression, which provided new insight into the role of ER in NAFLD.

*THY1* is another appealing protein, which is mostly involved in cell-cell and cell-matrix interactions, cell adhesion, migration, apoptosis, inflammation and fibrosis (34, 35). Emerging evidence has uncovered the relationship of *THY1* and ER stress. Transcriptional analysis from patients with cancers revealed that the expression of *THY1* was relevant to the activation of unfolded protein response (UPR), especially inositol-requiring enzyme 1α (IRE1α), one of the ER transmembrane proteins, suggesting a functional connections between *THY1* and ER (36).Previous studies revealed that the up regulation of *THY1* is responsible to alleviate interstitial pulmonary fibrosis *via* blockade of the WNT signaling pathway in acute interstitial pneumonia (37). Few studies on *THY1* in the field of NAFLD have been reported. In our study, we revealed that the expression of *THY1* exhibited an increasing trend with the progression of NAFLD, especially a strong value to indicate the severity of fibrosis (**Figure 4**), giving evidence to assume an important role of *THY1* in NAFLD. It would also be interesting to investigate whether *THY1* acts as a linker between ER stress and metabolic disorders in NAFLD.

Moreover, we identified several altered hepatic secretome genes in the progression of NAFLD. *IGFBP7* acts in response to growth hormone and involves in cell cycle progression, cell survival, migration and protein synthesis (38). The level of *IGFBP7* was increased significantly in the NAFLD progression. *COL1A1*, *COL1A2*, *COL3A1* and COL8A2 are response for fibrillar forming collagen and we have validated a higher circulated levels of collagen III in the MCD diet induced NASH mice model. Besides, the higher expression of *THY1* both in liver and circulation from NAFLD individuals indicate it a potential serum biomarker to suggest the severity of NAFLD. More hepatic secretory proteins identified in our study have not been reported to be associated with NAFLD yet. The exact physiological function and significance of hepatokines in NAFLD are interesting to be further explored.

In summary, our study provided a comprehensive understanding of ER function in the progression of NAFLD and provided promising biomarkers to evaluate the severity of NAFLD from a whole-transcriptome perspective. Further exploration should be carried out to add more evidence in understanding molecular mechanisms of liver diseases and offer valuable insights for future diagnostic and therapeutic applications.

## Data availability statement

The original contributions presented in the study are included in the article/**Supplementary Material**. Further inquiries can be directed to the corresponding authors.

## Ethics statement

The animal study was reviewed and approved by Ethics Committee of Sun Yat-sen University.

## Author contributions

YC and GS designed the study, reviewed the whole manuscript or granted financial support. RG and JW performed most experiment, conducted bioinformatic analysis and completed the manuscript. SL, XH, and ZR contributed to bioinformatic analysis and project design. HL, YL, and YZ contributed to review the whole manuscript. LZ, TW, JY, XX, SW, ZY, HA, and YW contributed to technical, material or administrative support. All authors contributed to the article and approved the submitted version.

## Funding

This work was supported by grants from the National Natural Science Foundation of China (81770826, 82070811), National Key R&D Program of China (2017YFA0105803), and Key Area R&D Program of Guangdong Province (2019B020227003).

## Acknowledgments

We wish to thank Yan Lu’s lab and Dr Bing Zhou for providing HFHC fed and normal control mice model sincerely.

## Conflict of interest

The authors declare that the research was conducted in the absence of any commercial or financial relationships that could be construed as a potential conflict of interest.

## Publisher’s note

All claims expressed in this article are solely those of the authors and do not necessarily represent those of their affiliated organizations, or those of the publisher, the editors and the reviewers. Any product that may be evaluated in this article, or claim that may be made by its manufacturer, is not guaranteed or endorsed by the publisher.

## Supplementary material

Supplementary Figure 1 Selection of ER related gene sets and sample clustering. **(A)** Upset plot summarizes the intersection among ER related gene sets. The bar plot in the left represents the gene number of each set. The lines in the right represent the intersection among each gene set. **(B)** Hierarchical clustering dendogram of the samples. The vertical axis represents the distance among samples to illustrate the clusters. The red line represents the height of 45000 to find out the obvious outliers.

## Abbreviations

AGPAT4, acylglycerophosphate acyltransferase 4; AH, alcoholic hepatitis; AUC, area under the curves; CERCAM, cerebral endothelial cell adhesion molecule, *COL1A1*, type I collagen alpha 1;*COL1A2*, type I collagen alpha 2; ER, Endoplasmic reticulum; ERAD, ER-associated degradation; *FKBP10* , FK506 binding protein 10; *IGFBP7*, Insulin-like growth factor-binding protein 7; IRE1α, inositol-requiring enzyme 1α; KEGG, Kyoto Encyclopedia of Genes and Genomes; MSigDB, Molecular Signature Database; MXRA8, matrix remodeling associated 8; NAFLD, non-alcoholic fatty liver disease; NAS, NAFLD activity score; PCA, principal component analysis; PPI, protein-protein interaction; *PTGDS*, prostaglandin D2 synthase; *QSOX1*, quiescin sulfhydryl oxidase homolog; *RHBDF1*, rhomboid family-1 gene; ROC, receiver operating characteristic; *STING1*, stimulator of interferon genes 1; *THY1*, thymocyte differentiation antigen 1; *TIMP1*, metallopeptidase inhibitor 1; TOM, topology overlay matrix; UPR, unfolded protein response; WGCNA, weighted gene co-expression network analysis.
