# Supplementary figures and images for: Comprehensive analysis of endoplasmic reticulum-related and secretome gene expression profiles in the progression of non-alcoholic fatty liver disease
Source: Front Endocrinol (Lausanne). 2022 Aug 12;13:967016. doi: 10.3389/fendo.2022.967016 (PMC9412753; doi:10.3389/fendo.2022.967016)

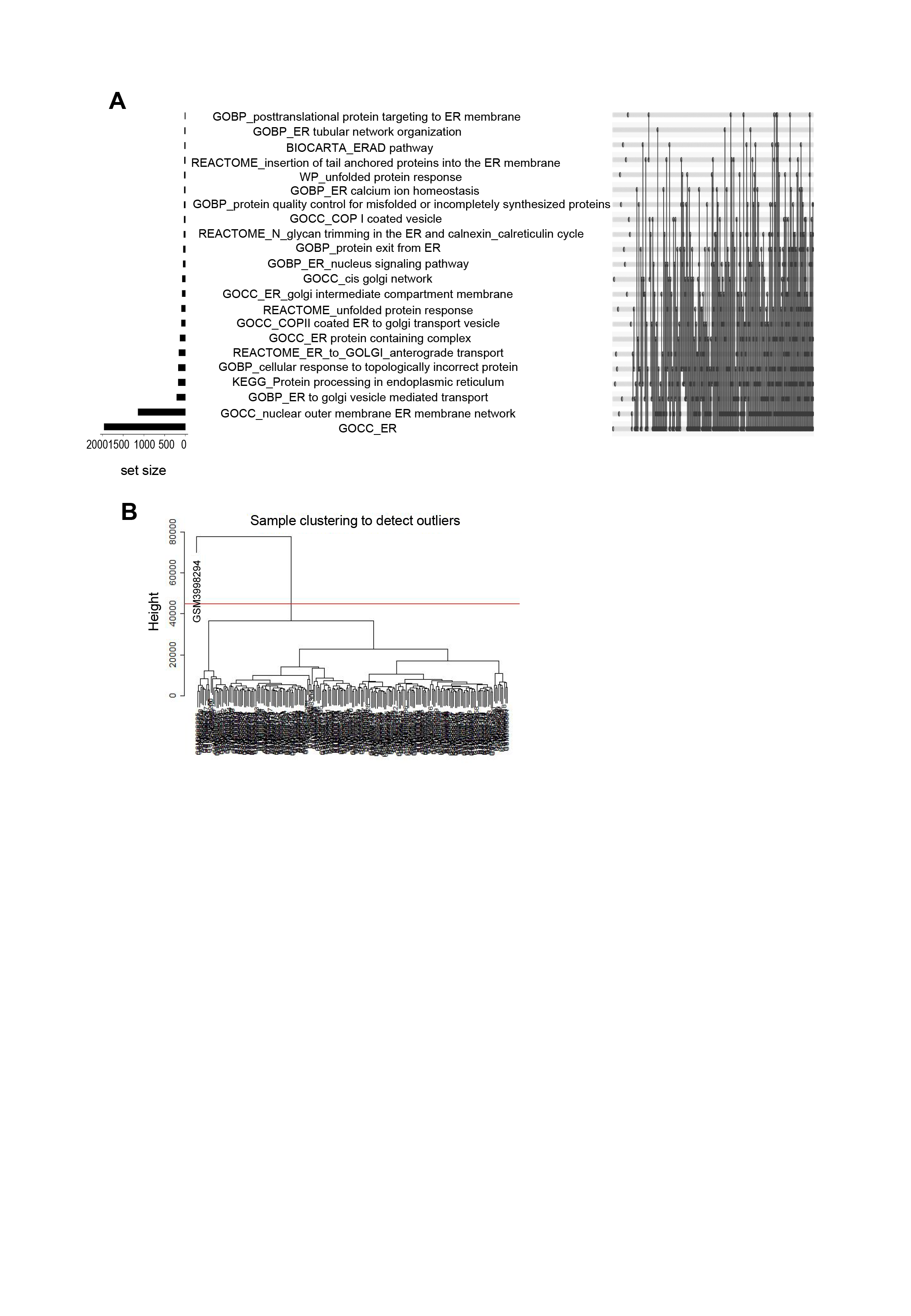

Supplement: Supplementary Figure 1 — Selection of ER related gene sets and sample clustering. (A) Upset plot summarizes the intersection among ER related gene sets. The bar plot in the left represents the gene number of each set. The lines in the right represent the intersection among each gene set. (B) Hierarchical clustering dendogram of the samples. The vertical axis represents the distance among samples to illustrate the clusters. The red line represents the height of 45000 to find out the obvious outliers. [file Image_1.tif]
